# 

**Published:** 1868

**Authors:** 


### CONTENTS.

### Number I.

### ORIGINAL COMMUNICATIONS.

PAGE.

ARTICLE I.—Chloride of Sodium. By A. Means, M. D., of Oxford, Ga., Professor of Chemistry in Atlanta Medical College, ... 1
ARTICLE II.—Medical Resources. By J. G. Westmoreland, M. D., Professor of Materia Medica in Atlanta Medical College, Atlanta, Ga ... 7
Atlanta Medical Society Reports ... 8
A Suspension of Splint, for Treating Simple and Compound Fractures of the Leg. By E. A. Clark, M. D., Resident Physician, St. Louis City Hospital ... 14
Cancer of Pylorus. By George P. Hannawalt, M. D., Washington, D. C ... 17
Successful Case of Teacheotomy in Croup. By J. H. Wythe, M. D., Salem, Oregon .. 20
Case of Nævus Successfully Treated by the application of Collodion. By L. S. Joynes, M. D., Professor of Physiology in the Medical College of Virginia. (Read before the Richmond Academy of Medicine) ... 21
Puffing of Doctors by the Newspapers ... 23
On Some Sources of Fallacy in the Diagnosis of Phthisis. By W. F. Wade, M. D., Physician to the General Hospital, Birmingham ... 26
On Sulphite of Magnesia By Joseph P. Remington, Brooklyn, N. Y ... 36
Syrupus Fresh Iodidi. By Edward R. Squibb, M.D ... 38
Senile Gangrene. By James T. Newman, M. D ... 40
On Gastrodynia. By W. H. Day, M. D, M. R. C. P., Physician to the Margaret Street Infirmary for Consumption and Diseases of the Chest ... 43
European Correspondence ... 54

### EDITORIAL AND MISCELLANEOUS.

Our Journal ... 55
Atlanta Medical College ... 57
Medical Association of Georgia ... 58
Married—Phares—Barfield ... 59
Bibliographical ... 60
Dr. Benjamin Dudley Lay ... 61
American Medical Association ... 61
Errata in January and February Nos ... 63

